# A randomized trial to evaluate attitudes regarding pharmacogenomics among pregnant and pediatric populations: design and baseline characteristics

**DOI:** 10.1038/s41397-026-00413-5

**Published:** 2026-04-23

**Authors:** Alexandra C. Sundermann, Salisha E. Marryshow Batson, Elizabeth A. Jasper, Sarah H. Jones, Darlene F. Fountain, Michelle Liu, Sudeep D. Sunthankar, Digna R. Velez Edwards

**Affiliations:** 1 Division of Quantitative and Clinical Sciences, Department of Obstetrics and Gynecology, Vanderbilt University Medical Center, Nashville, TN USA; 2 Institute for Population and Public Health, Vanderbilt University Medical Center, Nashville, TN USA; 3 Thomas P. Graham Jr. Division of Pediatric Cardiology and Center for Pediatric Precision Medicine, Department of Pediatrics, Monroe Carell Jr. Children’s Hospital at Vanderbilt and Vanderbilt University Medical Center, Nashville, TN USA; 4 Department of Pharmacy, Vanderbilt University Medical Center, Nashville, TN USA; 5 Department of Biomedical Informatics, Vanderbilt University Medical Center, Nashville, TN USA

**Keywords:** Genetics research, Predictive markers, Genetic testing, Predictive markers, Diagnostic markers

## Abstract

Pharmacogenomics (PGx) is a key component of precision medicine, yet most PGx studies exclude pregnant people and children. The VICE (Vanderbilt Integrated Center of Excellence)-MPRINT (Maternal and Pediatric Precision in Therapeutics): Maternal and Pediatric Pharmacogenetics Trial is a single-center, randomized control trial to assess how PGx testing and return of results influence patients’ understanding and perceived utility of PGx testing in a pregnancy and pediatric cohort. Pregnant individuals and families or guardians of children with a chronic health condition aged 0–16 were recruited. We assessed participants’ knowledge and attitudes regarding PGx testing at baseline and after receiving results with patient-friendly interpretations. Cases received an educational video about PGx testing with return of results. Controls received PGx test results alone. We aimed to determine perspectives regarding PGx in these understudied populations with the goal of promoting research and clinical implementation of PGx for pregnant women and children.

## Introduction

Pharmacogenomics (PGx) is a rapidly expanding field in translational research and precision therapeutics that began with the revelation that patient-level difference in drug response could be predicted based on specific, and often common, genetic variants. The goal of PGx testing is to personalize drug choice and dose; thereby allowing for increased drug efficacy and reduced adverse events, as suggested by specific gene-drug interactions [1, 2].

PGx testing to guide clinical prescribing of medications has recently become more accessible. There are now over 200 drugs with PGx information in the Food and Drug Administration (FDA) label and 75 drug-gene pairs designated by the Clinical Pharmacogenetics Implementation Consortium (CPIC) as clinically actionable [3–5]. However, studies regarding how PGx testing can guide medication or dose selection frequently exclude pediatric and pregnant populations, leading to inequity in who can benefit from the information gained from PGx testing [6]. Some of the medications identified by CPIC, such as 5-HT3 antagonists, selective serotonin reuptake inhibitors (SSRIs), and proton pump inhibitors, are commonly used in pregnant and pediatric populations, suggesting that these groups would also benefit from personalized guidance based on testing. PGx studies in pediatric and pregnant populations are crucial for establishing best practices in the utilization of PGx testing in these groups.

Pediatric and pregnant populations deserve thoughtful study that intentionally heeds the clinical and physiological considerations that are specific to these populations. There are dynamics in gene expression and pharmacokinetics that are unique to pregnancy and childhood [7–9], which makes the extrapolation of PGx-based clinical guidance established in the general adult population to pregnant individuals and children inappropriate. For example, cytochrome P450 2C19 (*CYP2C19*) expression is highly correlated with age during the first year of life, and therefore any clinical recommendations related to drugs that interact with *CYP2C19* in infants should account for age [10]. Previous work suggests differential induction of *CYP2D6* during pregnancy influences drug-gene associations, and therefore clinical guidance based on metabolizer status in pregnancy may differ from clinical guidance outside of pregnancy [11]. Study of drug-gene interactions in pregnant and pediatric populations is necessary to fully understand how genetic information can best guide clinical care [8, 12].

At the same time, there are special considerations when studying pediatric and pregnant populations that may make research specific to these groups more challenging. Additional regulatory oversight and ethical considerations are involved when studying pregnant or pediatric populations, making the approval and execution of these studies more complex and time intensive [13, 14]. The study of these populations is also subject to more nuanced temporal logistics. When studying pregnant populations, study protocol must account for the dynamic timeline of gestation. When studying pediatric populations, investigators must consider how physiology and gene-expression vary as children age, which may lead to complex eligibility determinations and scheduling of study events. Finally, securing consent for participation is also more complicated in pediatric and pregnant populations. In studies of pediatric populations, investigators must garner buy-in from families or guardians for consent for participation as well as assent from the child when possible [15]. Likewise, pregnant patients may be more cautious regarding study participation and more likely to seek partner approval before deciding to participate in a study during pregnancy than they would be outside of pregnancy [16]. On the other hand, some individuals cite the opportunity to get additional testing during pregnancy as motivation for study involvement [17]. Understanding perception of PGx testing and use in clinical care in pediatric and pregnant populations maybe helpful for promoting studies regarding PGx testing in these groups.

We aim to provide insight into the knowledge and attitudes regarding PGx testing in pregnant and pediatric populations with the goal of supporting research and clinical integration of PGx testing in the care of these patients. Using a community-engaged approach, we studied the knowledge and attitudes regarding PGx testing within these core populations at baseline and assessed how PGx testing and the return of results influences patients’ perceived utility of PGx testing.

### Objectives

The goals of this study were: (1) elucidate knowledge and attitudes regarding PGx in pregnant and pediatric populations and (2) assess the influence of two interventions on participants’ knowledge and attitudes regarding PGx testing. The first intervention was PGx testing and patient-friendly results and the second intervention was an educational video about PGx testing. Our primary hypothesis was that return of PGx test results would lead to a significant increase in knowledge and perceived utility of PGx testing. Our secondary hypothesis was that population-specific educational materials would further promote understanding and perceived value of PGx testing. We intend for this information to provide researchers and clinicians insight into these populations’ existing knowledge and views of PGx that can be used to support more equitable PGx research and integration in clinical care.

## Methods

### Maternal and pediatric precisions in therapeutics (MPRINT)

The Maternal and Pediatric Precision in Therapeutics (MPRINT) Hub is a national resource for conducting therapeutics-focused research with the aim to aggregate and expand the knowledge, tools, and expertise in maternal and pediatric therapeutics [18, 19]. MPRINT was formed out of the motivation to address significantly unmet needs relevant to the treatment of children across the spectrum of development and of individuals during pregnancy and the postpartum period.

This research was conducted out of the Vanderbilt Integrated Center of Excellence in MPRINT (VICE-MPRINT) at Vanderbilt University Medical Center (VUMC). The goal of this center is to conduct cutting-edge clinical, translational, basic, and data science research in maternal and pediatric therapeutics. This project specifically was designed to support precision medicine for maternal and pediatric care through PGx research.

### Study design and target populations

**Fig. 1:**
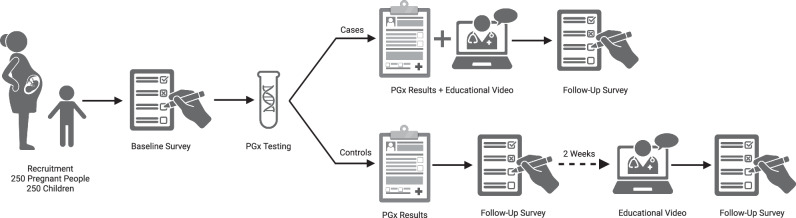
Overview of study design. Sequence of pharmacogenomic testing and survey administration were the same for the pediatric and pregnancy cohorts.

The VICE-MPRINT: Maternal and Pediatric Pharmacogenetics Trial is a single-center, two-armed randomized control trial (RCT) to assess the efficacy of two interventions on knowledge and attitudes regarding PGx testing among pregnant and pediatric populations (Fig. 1). This trial is registered with ClinicalTrials.gov (ID NCT05037305). In brief, two cohorts (a pregnancy cohort and a pediatric cohort) were recruited and we assessed baseline knowledge and attitudes regarding PGx testing. Then participants underwent PGx testing and received patient-specific interpretations of results. Interpretation of results were developed by the Pharmacogenetics Team within the PREDICT Program, which includes a dedicated pharmacogenetics pharmacist [20]. Standardized patient-friendly explanations have been developed for each possible phenotype associated with a gene-drug pair. The interpretation provided to the patients are individualized based on their specific phenotype. Cases also received an educational video further explaining PGx when they received their results, while controls received only their patient-specific interpretation of results initially. Knowledge and attitudes regarding PGx testing were again assessed to determine if the process of PGx testing and the return of test results with or without an additional educational video influenced participants’ perspectives. After the first follow-up survey was completed, study controls received the educational video after a two-week delay and were then administered the survey of knowledge and attitudes regarding PGx testing a third time. All surveys were administered electronically through REDCap [21].

### Eligibility

Full details regarding the inclusion and exclusion criteria for the pregnancy and pediatric cohorts are presented in Table 1. Common to both cohorts, individuals had to be registered patients at VUMC and possess a valid My Health at Vanderbilt (MHAV) account to be eligible. MHAV is a secure, online patient tool utilized by VUMC to communicate medical information, make appointments, and streamline communications between patients and providers or researchers. MHAV was utilized in this study to coordinate research events and to disseminate PGx testing results.

**Table 1:** VICE-MPRINT: maternal and pediatric pharmacogenetics trial inclusion and exclusion criteria.

Pregnancy Cohort	
Inclusion • Must be able to provide consent in English • Age 18 or older • Receive prenatal at VUMC • Currently 12 to 30 weeks gestational age • Completed or scheduled first prenatal visit at VUMC clinic • Intend to deliver at VUMC or affiliate • Agrees to receive findings from PGx testing • Allows access to their medical record	Exclusion • Stem cell or solid organ transplant • Blood or blood component transfusion within the previous 2 months • Inability to provide DNA sample for testing • Prior PGx testing • Use of in vitro fertilization (IVF) or assisted reproductive technologies (ART) to get pregnant, as they may have extensive experience/knowledge of genomics
Pediatric Cohort	
Inclusion • Must be able to provide consent (parent/guardian) and assent (child aged 7-16 years) in English, where possible. • Children aged 0 years to 16 years • Receive primary care or specialty care at VUMC • Must have a chronic health condition • Parent/guardian (for children aged 0-16 years) and child (age 7-16 years) must agree for both parent and child to receive findings from PGx testing • Parent/guardian (for children aged 0-16 years) and child (age 7-16 years) must allow study team to access to their medical record • Have a MHAV account or willing to be enrolled in MHAV	Exclusion • Stem cell or solid organ transplant • Blood or blood component transfusion within the previous 2 months • Inability to provide DNA sample for testing • Prior PGx testing

*MHAV* My Health at Vanderbilt, *PGx* pharmacogenomics, *VUMC* Vanderbilt University Medical Center.

### Recruitment

This study was conducted at VUMC in Nashville, Tennessee. Prior to onset of study recruitment, we conducted a community engagement studio to determine optimal recruitment methods for enrolling a diverse cohort of pregnant women and children and to inform design of survey tools that adequately capture participants’ perspectives [22].

Recruitment materials were circulated in VUMC primary care, obstetrics, and pediatric offices as well as other public locations at VUMC. Utilizing existing partnerships, study materials were also circulated through community organizations and health centers outside of VUMC to promote participation among underserved populations. The study was advertised through VUMC’s patient and research portals, through social media outreach on Facebook and X through Vanderbilt’s Research Distribution Listserv, and ResearchMatch, a collaborative project led by Vanderbilt Institute for Clinical & Translational Research.

### Screening and enrollment

Individuals interested in participation contacted study personnel through the email address or phone number provided on study promotional materials or by responding directly to a link in study materials distributed through email or MHAV messages. They then completed an electronic REDCap screening survey to determine eligibility (Appendix 1: Screening Survey for Pregnancy Cohort; Appendix 2: Screening Survey for Pediatric Cohort). Prospective participants were provided a dedicated study phone number to call if they had questions during the screening process. If eligibility criteria were met, consent was obtained from individuals (pregnancy cohort) or parents/guardians (pediatric cohort) for study participation. In cases where the child in the pediatric cohort was aged 7–16, assent for study participation was also obtained. Consented participants received a phone call by the research staff to provide details about scheduling PGx testing and to answer questions about the study protocol.

### Baseline demographic survey

Study participants completed baseline surveys prior to randomization (Appendix 3: Baseline Questionnaire for Pregnancy Cohort; Appendix 4: Baseline Questionnaire for Pediatric Cohort). Surveys were self-administered online through REDCap. For the pediatric cohort, survey items were tailored to reflect parental or guardian completion of survey with child input when applicable. In the baseline survey participants provided demographic data, health, and lifestyle information. Participants in the pregnancy cohort also provided information regarding obstetric history.

### Knowledge and attitudes regarding pharmacogenomic testing

Participants completed an assessment of baseline knowledge and attitudes regarding PGx testing. Prior to survey development, we performed a literature review of studies of PGx surveys and adapted language for relevant survey items for our target audience [23–37]. The survey captured participants’ responses in the following core domains: (1) experience with medications and adverse events; (2) understanding of genetics and PGx; (3) impact of PGx on relationships with healthcare professionals; (4) scenarios involving PGx-guided prescribing; (5) perceptions of the utility of PGx information; and (6) responses to PGx educational materials (domain 6 on follow-up survey only). Questions specific to perceptions and knowledge were scored on a 5-point Likert scale (e.g. “very important” to “not at all important”; or “very likely” to “not likely”). The language and format of the questions were modified for the pediatric cohort to reflect parental/guardian completion of survey with input from the child.

### Biospecimen sample collection and pharmacogenomics

DNA samples were collected via blood draw at a VUMC lab during a scheduled appointment. The VUMC Molecular Diagnostic lab running the sample is Clinical Laboratory Improvement Amendments (CLIA) and Certified Administrative Professional (CAP) certified. The PGx panel detects 37 unique PGx variants in 10 genes using a QuantStudio^TM^ 7 Pro Real-Time PCR System and a custom designed TaqMan^TM^ Array Card (ThermoFisher Scientific) (Appendix 5: Pharmacogenetic Testing Panel) [38]. A separate TaqMan® Copy Number assay for *CYP2D6* was performed for each sample using a 96-well plate format.

### Randomization and study interventions

We aimed to determine the impact of two interventions on knowledge and attitudes regarding PGx testing. The first intervention was the PGx testing and return of results and the second intervention was an educational video made by the study team. PGx test results were sent to participants through the MHAV patient portal. Results were accompanied by patient-friendly explanations for interpretation of results. PGx results were also uploaded to the participant’s VUMC’s electronic medical health record. The study team created an educational video to aid in the interpretation of PGx test results (Appendix 6: Educational Video Script).

To study the influence of these two interventions on knowledge and attitudes regarding PGx testing, participants were randomized into two arms. Randomization was performed at the time of study enrollment through a built-in feature of REDCap. Cases received PGx test results and the educational video simultaneously and controls received the PGx results then the educational video two weeks later, with surveys at each time point. Test results included an individualized report of participant metabolizer status as indicated by each variant tested and a list of corresponding medications. These test results were also made available in the participants’ electronic health record. Participants were administered the same survey about knowledge and attitudes regarding PGx testing they received at enrollment with the addition of several items specific to educational materials provided after testing. Surveys were to be completed within two weeks of receiving PGx test results. Controls were then provided the educational video two weeks from the completion of the first survey and were then administered the survey about knowledge and attitudes regarding PGx testing a third time. If participants did not complete the follow-up survey after six phone or email reminders from study personnel, they were considered lost to follow-up.

### Analysis plan and power calculations

We aimed to recruit 250 pregnant women and 250 children. Assuming equal randomization and an α = 0.05, we will have 80% power to detect an effectiveness of study intervention in participant-reported improved understanding of PGx testing for a risk ratio of a magnitude of 1.35 or higher (Appendix 7: Power Calculation).

Data from the pregnancy and pediatric cohorts were analyzed separately. For both cohorts, baseline characteristics of participants were described using medians and inter-quartile ranges for continuous variables and counts and proportions for categorical variables. Not all individuals who were enrolled and randomized in the study followed through with PGx testing. Therefore, we also use logistic regression to present bivariate analysis for the association between baseline maternal demographics and PGx testing follow through.

## Results

### Recruitment

**Fig. 2:**
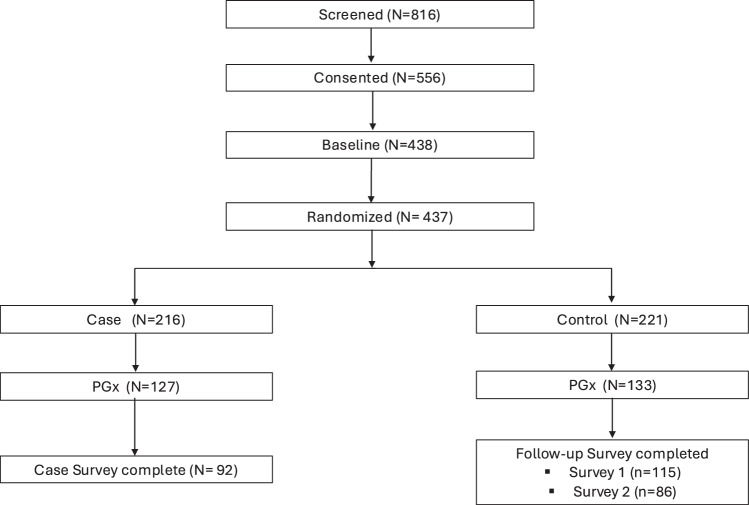
Pregnancy Cohort Identification. Flow diagram demonstrates selection of pregnancy cohort including exclusion criteria, randomization, and final sample size.

**Fig. 3:**
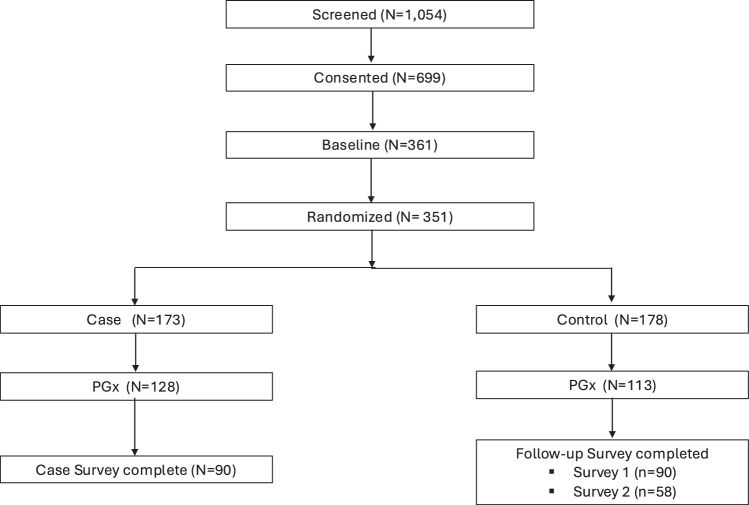
Pediatric Cohort Identification. Flow diagram demonstrates selection of pediatric cohort including exclusion criteria, randomization, and final sample size.

Trial enrollment for the pregnancy cohort was started July 2022 and was completed October 2024. Enrollment for the pediatric cohort was started July 2023 and was completed December 2025. During recruitment, 816 pregnant women were screened and 437 were randomized and participated in the trial (Fig. 2). 1054 pediatric patients were screened and 351 were randomized for trial participation (Fig. 3).

### Characteristics of cohorts and PGx testing

Among the individuals who were fully enrolled in the pregnancy cohort (*n* = 437), median age was 30 years old (inter-quartile range 26, 34) and 80.7% of participants identified as non-Hispanic White, 4.5% identified as non-Hispanic Black, and 7.3% identified as Hispanic or Latina. Over half of participants had a Bachelor’s or graduate degree (52.2%). Over eighty percent of participants described their health as “good or very good,” 67.5% of participants reported taking at least one medication daily, and 44.4% of participants reported experiencing an adverse medication side-effect at some point in life. Of the participants enrolled and randomized in the study, 260 followed through with PGx testing (59.7%). Participants who were older, had a higher education attainment, were from higher income households, or reported taking a medication regularly were more likely to follow through with PGx testing. Participants who identified as Hispanic, who were single, or who had public insurance were less likely to follow through with PGx testing (Table 2).

**Table 2:** Pregnancy cohort characteristics by PGx testing status^a^.

	PGx Testing (*n* = 260)		No PGx Testing (*n* = 177)		OR (95% CI)
Characteristic	N	%	N	%	
Maternal age, years					
<25	39	15.00	46	25.99	Referent
25–29	68	26.15	49	27.68	1.64 (0.93, 2.87)
30–34	85	32.70	52	29.38	1.93 (1.11, 3.34)
≥ 35	68	26.15	30	16.95	2.67 (1.46, 4.90)
Race					
Black	15	5.77	9	5.08	1.12 (0.48, 2.62)
Other	16	6.15	14	7.91	0.89 (0.30, 2.62)
White	221	85.00	148	83.62	Referent
Prefer not to answer	8	3.08	6	3.39	–
Ethnicity					
Hispanic or Latino	13	5.00	19	10.73	0.43 (0.20, 0.89)
Not Hispanic or Latino	244	93.85	152	85.88	Referent
Prefer not to answer	3	1.15	6	3.39	–
Education					
High school or less	36	13.85	50	28.25	Referent
Some education after high school	70	26.92	51	28.81	1.91 (1.09, 3.34)
Bachelor’s degree	66	25.39	46	25.99	1.99 (1.13, 3.52)
Graduate degree	87	33.46	29	16.38	4.17 (2.29, 7.59)
Unknown or prefer not to answer	1	0.38	1	0.57	–
Household income					
≤ $50,000	62	23.84	54	30.51	Referent
$50,001–$75,000	34	13.08	39	22.03	0.76 (0.42, 1.37)
$75,001–$100,000	34	13.08	16	9.04	1.85 (0.92, 3.72)
> $100,000	114	43.85	47	26.55	2.11 (1.28, 3.48)
Prefer not to answer	16	6.15	21	11.87	–
Marital status					
Married	196	75.39	110	62.15	Referent
Single	45	17.31	54	30.51	0.47 (0.30, 0.74)
Other	16	6.15	11	6.21	1.46 (0.51, 4.20)
Prefer not to answer	3	1.15	2	1.13	–
Gravida					
Only current pregnancy	82	31.54	58	32.77	Referent
Twice	65	25.00	43	24.29	1.07 (0.54, 1.78)
Three times	48	18.46	28	15.82	1.21 (0.68, 2.15)
Four or more	64	24.62	46	25.99	0.98 (0.59, 1.63)
Missing	1	0.38	2	1.13	–
Health insurance coverage					
Private (1–3)	165	63.46	93	52.54	Referent
Public (4–6)	84	32.31	73	41.24	0.64 (0.43, 0.95)
Unknown, multiple, or none	11	4.23	11	6.22	–
Smoking in pregnancy					
No	242	93.08	164	92.66	Referent
Yes	15	5.77	13	7.34	0.78 (0.36, 1.69)
Prefer not to answer	3	1.15	0	0.00	–
Self-reported health					
Poor	0	0.00	0	0.00	–
Fair	26	10.00	11	6.21	1.38 (0.64, 2.97)
Good	110	42.31	64	36.16	Referent
Very Good	97	37.31	82	46.33	0.69 (0.45, 1.05)
Excellent	26	10.00	18	10.17	0.84 (0.43, 1.65)
Prefer not to answer	1	0.38	2	1.13	–
Regular medication use					
Yes	188	72.31	107	60.45	1.76 (1.17, 2.64)
No	70	26.92	70	39.55	Referent
Prefer not to answer	2	0.77	0	0.00	–
History of medication adverse effect					
Yes	113	43.46	81	45.76	0.90 (0.60, 1.34)
No	127	48.85	82	46.33	Referent
Prefer not to answer	20	7.69	14	7.91	–

*PGx* pharmacogenomics.

^a^This table compares enrolled study participants who followed through with PGx testing to those who did not follow through with testing.

Individuals who were fully enrolled in the child cohort (*n* = 361) also predominantly identified as non-Hispanic White (77.8%). Approximately 5.8% of the cohort identified as non-Hispanic Black, and 7.8% identified as Hispanic or Latino. Almost 63% of children were six years old or younger. This matched the educational attainment of children, as 54.6% had no education and 39.9% were in elementary school (kindergarten to 5th grade). Roughly 80% of child participants reported being in “good, very good, or excellent health.” Taking at least one medication daily was reported among 86.7% of participants and 32.1% reported experiencing an adverse medication side-effect at some point. Of the 361 who enrolled in the study and were randomized, 241 followed through and received PGx testing (66.8%). Participants who were six years old or younger, whose parents or guardians were single, or who had public insurance were less likely to follow through with PGx testing (Table 3).

**Table 3:** Children cohort characteristics by PGx testing status^a^.

	PGx Testing (*n* = 241)		No PGx Testing (*n* = 120)		OR (95% CI)
Characteristic	N	%	N	%	
Child sex					
Female	103	42.74	51	42.50	1.02 (0.65, 1.59)
Male	137	56.85	69	57.50	Referent
Prefer not to answer or unknown	1	0.41	0	0.00	
Child age, years					
0–6	141	58.51	86	71.67	Referent
7–11	80	33.19	27	22.50	1.80 (1.09, 3.04)
12–16	20	8.30	7	5.83	1.72 (0.72, 4.59)
Race					
Black	16	6.64	7	5.83	1.07 (0.44, 2.91)
Other	25	10.37	18	15.00	0.66 (0.34, 1.29)
White	200	82.99	95	79.17	Referent
Ethnicity					
Hispanic or Latino	19	7.88	9	7.50	1.05 (0.47, 2.52)
Not Hispanic or Latino	218	90.46	109	90.83	Referent
Unknown or prefer not to answer	4	1.66	2	1.67	–
Education					
None	125	51.88	72	60.00	Referent
Elementary (Kindergarten-5th grade)	102	42.32	42	35.00	1.40 (0.88, 2.23)
Middle (6th–8th grade)	13	5.39	3	2.50	2.40 (0.73, 11.22)
High school	0	0.00	2	1.67	-
Unknown or prefer not to answer	1	0.41	1	0.83	–
Household income					
≤ $50,000	55	22.82	49	40.83	Referent
$50,001–$75,000	45	18.67	20	16.67	1.99 (1.04, 3.89)
$75,001–$100,000	43	17.85	19	15.83	2.00 (1.04, 3.96)
>$100,000	73	30.29	23	19.17	2.80 (1.54, 5.22)
Prefer not to answer	25	10.37	9	7.50	–
Marital status					
Married	185	76.76	78	65.00	Referent
Single	31	12.86	29	24.17	0.45 (0.25, 0.80)
Other	21	8.72	12	10.00	0.74 (0.35, 1.62)
Prefer not to answer	4	1.66	1	0.83	–
Health insurance coverage					
Private (1–3)	105	43.57	35	29.17	Referent
Public (4–6)	96	39.83	69	57.50	0.47 (0.28, 0.76)
Unknown, multiple, or none	40	16.60	16	13.33	0.83 (0.42, 1.70)
Self-reported health					
Poor	7	2.91	1	0.83	3.34 (0.56, 86.48)
Fair	35	14.52	24	20.00	0.78 (0.43, 1.45)
Good	112	46.47	60	50.00	Referent
Very Good	69	28.63	30	25.00	1.23 (0.73, 2.11)
Excellent	15	6.22	5	4.17	1.57 (0.57, 5.14)
Prefer not to answer	3	1.25	0	0.00	–
Regular medication use					
Yes	215	89.21	98	81.67	1.93 (1.03, 3.60)
No	25	10.37	22	18.33	Referent
Prefer not to answer	1	0.41	0	0.00	–
History of medication adverse effect					
Yes	88	36.51	28	23.33	2.09 (1.26, 3.51)
No	123	51.04	82	68.33	Referent
Prefer not to answer	30	12.45	10	8.33	–

*PGx* pharmacogenomics.

^a^This table compares enrolled study participants who followed through with PGx testing to those who did not follow through with testing.

### Knowledge and attitudes of pharmacogenomics at baseline among participants in the pregnancy cohort

**Fig. 4:**
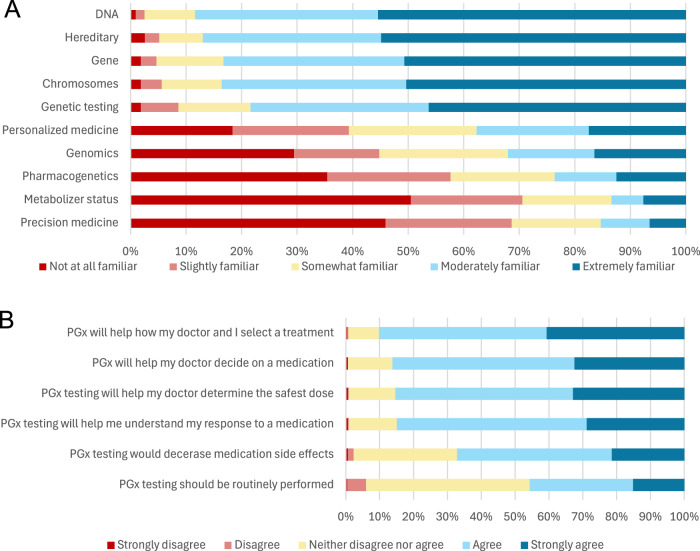
Familiarity with and attitudes towards pharmacogentic testing at baseline among the pregnancy cohort. Familiarity with key pharmacogenetic testing terminology among participants in the pregnancy cohort at baseline (**A**; N = 437). Attitudes about the utility of pharmacogenetic testing among participants in the pregnancy cohort at baseline (**B**; N = 437). Participants reported familiarity with terms on a five-point Likert scale. Each bar shows the distribution of participants’ responses and totals to 100%.

Most participants believed that genes contributed to how individuals respond to medications (90.4%). Almost all participants were familiar with basic terms like DNA, chromosomes, and genes, but fewer reported familiarity with more complex terminology, including pharmacogenetics, metabolizer status, and precision medicine (Fig. 4A). After being provided with a definition of PGx testing, 90.2% of individuals “agreed” or “strongly agreed” that PGx would help how they and their doctor select a treatment. Most participants “agreed” or “strongly agreed” that PGx testing would help their doctor decide on a medication that is most likely to treat their illness (86.2%), help their doctor determine the safest dose (85.3%), help them understand why they or a family member did not tolerate or respond to specific medications (84.8%), and decrease the risk of experiencing an adverse medication side effect (67.1%). Less than half of participants thought that PGx testing should be routinely performed (45.6%; Fig. 4B).

### Knowledge and attitudes of pharmacogenomics at baseline among participants in the pediatric cohort

**Fig. 5:**
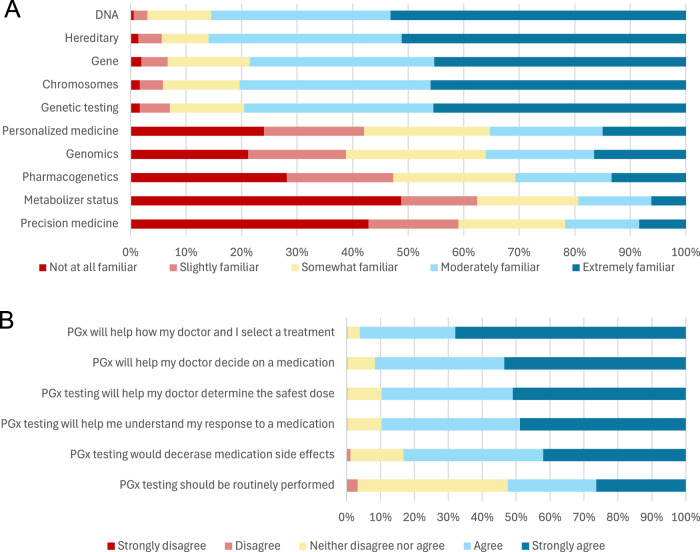
Familiarity with and attitudes towards pharmacogentic testing at baseline among the pediatric cohort. Familiarity with key pharmacogenetic testing terminology among parents/guardians of participants in the pediatric cohort at baseline (**A**; N = 361). Attitudes about the utility of pharmacogenetic testing among participants in the pregnancy cohort at baseline (**B**; N = 361). Participants reported familiarity with terms on a five-point Likert scale. Each bar shows the distribution of participants’ responses and totals to 100%.

Most families/guardians of participants in the pediatric cohort believed that genes contributed to how individuals respond to medication (86.1%). Almost all individuals were at least somewhat familiar with basic terms (DNA, chromosomes, genes), but there was less familiarity ( < 50%) with more complex terminology, such as metabolizer status and precision medicine (Fig. 5A). After participants were provided with a definition for PGx testing, almost all (96.1%) individuals “agreed” or “strongly agreed” that test results would help them and their doctor make decisions about treatment. Most also “agreed” or “strongly agreed” that PGx testing would help their doctor decide what medication would be the best to treat their illness (91.6%), help their doctor determine the safest dose (89.6%), help them understand why they or a family member did not tolerate or respond to specific medications (89.7%), and decrease the risk of experiencing an adverse medication side effect (83.2%). About half of families/guardians of participants in the pediatric cohort thought that PGx testing should be routinely performed (52.5%; Fig. 5B).

## Discussion

PGx is a foundational component of precision medicine, yet pregnant and pediatric populations are frequently excluded from PGx studies. Without studies that assess the role of PGx in the context of the unique pharmacokinetics observed in these populations, we cannot establish best evidence-based practices. We established two cohorts to illuminate knowledge and attitudes of PGx testing in these populations with the goal of informing future PGx research and clinical implementation. On baseline surveys, most participants in both the pregnancy and pediatric cohorts were not familiar with terminology related to PGx testing and precision medicine. However, after being provided with a brief definition of PGx, over 90% of participants had a favorable impression of its utility for guiding clinical care.

The majority of PGx studies exclude pregnant women and children, so the characterization of these known gene-drug interactions has not been verified in these populations. Pregnant and pediatric populations are intrinsically distinct pharmacokinetically from the general population. Individual differences in drug response are in part driven by genetic differences in enzymes, transporters, drug binding, drug receptors, signaling molecules, and other molecular targets [7]. While many gene-drug interactions have been established, these interactions in the unique pharmacodynamic setting of either childhood or pregnancy are not well understood and existing PGx tests, including the panel utilized in this study, lack evidence for clinical use in these populations.

In the community-based cohort of pregnant patients, we found that a general awareness of PGx was limited. In the baseline interview, over half of participants in the pregnancy cohort were unfamiliar with the terms “genomics,” “pharmacogenetics,” “metabolizer status,” and “precision medicine.” However, after patients were provided a simple definition of PGx as “the use of difference in your DNA to choose the right medication or dose,” over 90% of participants reported believing this information would be useful for their doctors to make the best decision for them. Similar baseline attitudes and perceptions were reflected in the responses of families/guardians of participants in the pediatric cohort. This suggests that familiarity with PGx testing and its utility is a barrier to its application in these populations, but one that may be easily overcome.

With the development of these cohorts, we conducted a community engagement studio to best understand recruitment strategies for enrolling diverse cohorts. We distributed recruitment materials in a wide variety of clinical settings and through our community outreach partners with this goal in mind. Despite these efforts, a high proportion of participants in both cohorts identified as non-Hispanic, White. Further, among participants enrolled and randomized in both cohort, participants who were a part of a racial or ethnic minority group or who had public insurance were less likely to follow through with PGx testing. While this analysis benefits from having information about baseline perceptions and attitudes regarding PGx testing from the full set of enrolled participants, our future analyses focused on how study interventions (including PGx testing and return of results) influence perceptions and attitudes will have to account for differential participation in study events by baseline characteristics.

Pregnant and pediatric populations are subject to unique pharmacokinetic dynamics that are not adequately reflected in the general adult population. Best practices for the use of PGx will remain obscure until we study known drug-gene interactions in these contexts. In the analysis of baseline perceptions of both cohorts, we found that pregnant patients and families of pediatric patients were open to the integration of PGx testing in their care. Thus, patient acceptance is not anticipated to be a meaningful barrier to the study of PGx testing and application in these populations. Next, we aim to assess how the return of PGx test results and educational materials influences participants’ perceptions in both cohorts. By better understanding knowledge and attitudes regarding PGx in these populations, we can identify paths towards more equitable PGx research and implementation for all.

## Supplementary information

Appendix 1

Appendix 2

Appendix 3

Appendix 4

Appendix 5

Appendix 6

Appendix 7

## Data Availability

The datasets generated during this analysis (de-identified characteristics of study participants and responses to surveys on attitudes regarding pharmacogenetic testing) are available from the corresponding author on reasonable request after completion of institutionally-mandated data use agreement.
