# Supplementary material for: A randomized trial to evaluate attitudes regarding pharmacogenomics among pregnant and pediatric populations: design and baseline characteristics
Source: Pharmacogenomics J. 2026 Apr 23;26(3):16. doi: 10.1038/s41397-026-00413-5 (PMC13106030; doi:10.1038/s41397-026-00413-5)
Supplement: Supplementary file 2 — Appendix 2 [file 41397_2026_413_MOESM2_ESM.pdf]

Screening for Parents and Children

Thank you for your interest in the study. You and your child are being asked to take part in this research screening activity to determine whether you are eligible for a study to learn more about what parents of children with chronic health conditions think about genetic (DNA) tests that tell you and your child how your child's body uses and processes different medicines.

This screening is for research purposes to determine if you are eligible to participate in the study. The purpose of the main study is to look at genes (DNA) and how they affect medication response. Genes are the instruction manual for your body. The genes you get from your parents decide what you look like and how your body behaves. The genes we are testing in this study can help us predict how your child may respond to certain medicines. The research activities in the main study include a blood draw for your child and a survey to be completed by you (with the help of your child if they are old enough).

This form provides information on the screening activity (survey). If you are eligible to participate in the main study, you will be asked to sign a separate consent form with more information on the main study. This screening activity includes completing a survey which will take about 10 minutes. Risks of participating in the screening survey include possible breach of confidentiality, but there are many safeguards in place to prevent the release of this information including secure data storage through this survey platform. There are no direct benefits to you for completing this screening survey.

There is no cost to you for participating in this screening activity and you will not be compensated for completing the screening survey. Compensation is offered for the main study as described in the main consent form.

Participation in this screening activity is voluntary and you may choose not to participate. Refusal to participate in this screening activity will involve no penalty or loss of benefits to which you may be otherwise entitled, and you may discontinue participation at any time without penalty.

If you have any questions or would like to withdraw your consent to use your survey information, please contact Dr. Digna Velez Edwards at 615-875-4491 or by email at pgx-survey@vumc.org. You may also contact her by mail at VICE-MPRINT Study, Department of Pediatrics, 2141 Blakemore Avenue, Nashville, TN 37212. For additional information about your rights as a person taking part in this screening, to discuss problems, concerns, and questions, or to offer input, please feel free to call the VUMC Institutional Review Board Office at (615) 322-2918 or toll free at (866) 224-8273.

Your screening information, with or without identifiers, will not be shared or distributed for future studies. If you are not eligible for the main study, we will destroy your responses.

Do you want to continue to the screening questions to see if you and your child can be in this study? We will not keep any information about you if you do not want to continue with the screening.	◯ Yes<br>◯ No

Thank you for taking the time to see if you and your child are eligible for this study. If you are eligible, you will have the opportunity to download and read the consent form for information about your rights as a research participant. We will also ask you and your child to do a short survey.

Please answer a few short questions to help us determine whether you and your child are eligible to participate:

Is your child between the ages of 0 and 16 years old?	◯ Yes<br>◯ No
How old is your child, in years?	◯ 0 - 6 years<br>◯ 7 - 11 years<br>◯ 12 - 16 years
Does your child receive primary or specialty care at Vanderbilt?	◯ Yes<br>◯ No

Does your child have a My Health At Vanderbilt account, or are you willing to get one for your child?	◯ Yes<br>◯ No
Does your child have a chronic health condition?	◯ Yes<br>◯ No
Are you and your child willing to have a tube of blood drawn from your child for this study?	◯ Yes<br>◯ No
Are you and your child willing to have a DNA test done on your child?	◯ Yes<br>◯ No
Are you and your child willing to have the results of the DNA test returned to you?	◯ Yes<br>◯ No
Are you willing to have the results of your child's DNA test put in their medical record?	◯ Yes<br>◯ No
Do you allow us access to your child's medical record?	◯ Yes<br>◯ No
Has your child ever had a stem cell transplant or a solid organ transplant?	◯ Yes<br>◯ No
Has your child had a blood or blood component transfusion in the previous 2 months?	◯ Yes<br>◯ No
Have you or your child ever had DNA testing of your blood or cells for the purpose of medication prescribing before?	◯ Yes<br>◯ No

You are eligible to participate in this study. Please provide your contact information below. We will use this information to contact you regarding the study but will not keep it should you later decide not to participate in the study.

Child's First Name	______________________
Child's Last Name	______________________
What is your child's date of birth?	______________________
I would prefer to be contacted via	◯ Phone<br>◯ Email
Phone Number	______________________
Is it okay to leave a voicemail at this number?	◯ Yes<br>◯ No

Okay, we will not leave a voicemail.

Email

____

Thank you for answering the questions. Your child is eligible to participate in this study.
Please call us at 615-875-4491 to discuss the study and to give us a chance to answer any questions or address any concerns you may have before proceeding. If you don't reach us, please wait for a call back before continuing. You can save the survey at any time with the "Save and Return Later" button. We will try to return all calls within 24 hours (Monday through Friday).

Please continue the survey to the next section. It contains the Consent form you will need to read and sign electronically. You can also download and print the consent form.

Being in this study is you and your child's choice. You and your child can decide not to be in this study at any time. Deciding to not be part of the study will not change your regular medical care in any way. If you and your child decide to stop being part of the study, you should tell the study doctors.

This is a study being conducted by researchers at Vanderbilt University Medical Center. You and your child are being asked to participate and provide a blood sample to look at your child's genes (DNA). If you and your child participate in this study, the blood sample and your child's DNA will be used as part of this study.

Unfortunately, your child is not eligible to participate in this study.

We appreciate your interest in this study and the time you took to complete the screening questions.

Thank you for your time.
