# Supplementary material for: A randomized trial to evaluate attitudes regarding pharmacogenomics among pregnant and pediatric populations: design and baseline characteristics
Source: Pharmacogenomics J. 2026 Apr 23;26(3):16. doi: 10.1038/s41397-026-00413-5 (PMC13106030; doi:10.1038/s41397-026-00413-5)
Supplement: Supplementary file 3 — Appendix 3 [file 41397_2026_413_MOESM3_ESM.pdf]

Initial Study Survey

Please complete the survey below.

Thank you!

The next few questions will ask for your contact information and how you would like us to stay in touch.

First Name __________________________________

Last Name __________________________________

Street __________________________________

City __________________________________

State __________________________________

Zip Code __________________________________

Phone Number __________________________________

Is is okay to leave a voicemail at this number?
- ◯ Yes
- ◯ No

Okay, we will not leave a voicemail.

Email __________________________________

The following questions will help us describe who participates in the study. You do not have to answer questions that make you feel uncomfortable.

What is your date of birth? __________________________________

What is your ethnicity?
- ◯ Hispanic or Latino
- ◯ NOT Hispanic or Latino
- ◯ Unknown
- ◯ Prefer not to answer

What is your race? Please check all that apply.	☐ American Indian or Alaska Native<br>☐ Asian<br>☐ Black or African American<br>☐ Native Hawaiian or Other Pacific Islander<br>☐ White or European American<br>☐ More Than One Race<br>☐ Unknown<br>☐ Prefer not to answer
What sex were you assigned at birth, on the original birth certificate?	◯ Female<br>◯ Male<br>◯ Intersex<br>◯ Prefer not to answer
What is your current gender identity?	◯ Female<br>◯ Male<br>◯ Transgender<br>◯ Do not identify as female, male, or transgender<br>◯ Prefer not to answer
What is the highest grade or level of education you have completed?	◯ Less than a high school graduate<br>◯ High school graduate or GED<br>◯ Some education after high school (technical/vocational school or some college)<br>◯ Bachelor's degree or equivalent<br>◯ Master's degree (MS, MBA, MFA, etc.)<br>◯ Doctoral or other professional degree (PhD, MD, JD, or other)<br>◯ Prefer not to answer
What is your current marital status?	◯ Single<br>◯ Married or living as married<br>◯ Widowed<br>◯ Divorced<br>◯ Separated<br>◯ Other<br>◯ Prefer not to answer
Including yourself, how many people currently live in your household	◯ 1<br>◯ 2<br>◯ 3<br>◯ 4<br>◯ 5<br>◯ 6<br>◯ 7<br>◯ 8<br>◯ 9<br>◯ 10+
How religious do you consider yourself to be?	◯ Not religious at all<br>◯ Not very religious<br>◯ Somewhat religious<br>◯ Very religious<br>◯ Prefer not to answer
What is your current work situation?	◯ Working<br>◯ Retired<br>◯ Disabled (permanently or temporarily)<br>◯ Other<br>◯ Prefer not to answer

What is your household's total combined income (pre-taxes) during the past 12 months?

- ◯ Less than $25,000
- ◯ $25,000 to $50,000
- ◯ $50,000 to $75,000
- ◯ $75,000 to $100,000
- ◯ $100,000 or more
- ◯ Don't know
- ◯ Prefer not to answer

Do you have health insurance or a health coverage plan? Please check all that apply

- ☐ Yes, through my employer
- ☐ Yes, through someone else's employer
- ☐ Yes, a plan that I or someone else buys
- ☐ Yes, through Medicare
- ☐ Yes, through Medicaid or Medical Assistance
- ☐ Yes, through the military, CHAMPUS, or the VA
- ☐ No, I don't have any coverage
- ☐ Don't know
- ☐ Prefer not to answer

How did you hear about the study?

- ☐ Vanderbilt email
- ☐ Research Match email
- ☐ Doctor or clinic told me about the study
- ☐ Letter or flyer in clinic
- ☐ Word of mouth
- ☐ Other

Other: ______________________

The following questions will help us understand your health history and your family health history. If family history information is unavailable for any reason, please mark "unsure" or "don't know." You do not have to answer questions that make you uncomfortable.

How tall are you (in feet and/or inches)? ______________________

How much did you weigh (in pounds) before you became pregnant? ______________________

In general, would you say your health is...

- ◯ Excellent
- ◯ Very good
- ◯ Good
- ◯ Fair
- ◯ Poor

Do any diseases, like heart disease, cancer, or diabetes, run in your family?

- ◯ Yes
- ◯ No
- ◯ Unsure
- ◯ Prefer not to answer

Do you think any of the diseases are caused by genes?

- ◯ Yes
- ◯ No
- ◯ Unsure
- ◯ Prefer not to answer

What disease do you believe is caused by a gene? ______________________

Do you have any of the following chronic conditions? Please check all that apply.

- ☐ Heart or vascular condition (Abnormal heart rhythm, high blood pressure, or other conditions affecting the heart or blood vessels)
- ☐ Lung condition (Asthma, COPD, or other conditions that affect breathing)
- ☐ Gastrointestinal or liver condition (Acid reflux, Crohn's disease, Celiac disease, hepatitis, or other conditions affecting your digestion or how your liver works)
- ☐ Kidney condition (Kidney stones, chronic kidney disease, and other conditions affecting how your body makes urine)
- ☐ Neurologic condition (Seizures, headaches, strokes, sleep disorders, and other conditions that affect the brain, spinal cord, and neurons)
- ☐ Conditions affecting your immune system (Immune deficiency, HIV/AIDS and other long-term viral, bacterial, fungal or other infections)
- ☐ Rheumatologic or autoimmune disease (Lupus, rheumatoid arthritis, and other conditions due to the immune system)
- ☐ Endocrine condition (Thyroid disease, diabetes, and other conditions affecting hormones)
- ☐ Hematologic (blood cell) condition (Conditions affecting red blood cells (anemia, thalassemia), white blood cells, or platelets)
- ☐ Skin condition (Atopic dermatitis, psoriasis, rosacea, and other conditions affecting the skin)
- ☐ Cancer (Cancer of any type, now or in the past)
- ☐ Behavioral or mental health condition (Depression, anxiety, PTSD, and other conditions affecting mental health)
- ☐ Reproductive or gynecological condition (Polycystic ovarian syndrome, endometriosis, or other conditions affecting the ovaries, uterus, or vagina)
- ☐ Other

Please list other chronic diseases you have

____

How far along is your pregnancy

- ◯ 12 weeks
- ◯ 13 weeks
- ◯ 14 weeks
- ◯ 15 weeks
- ◯ 16 weeks
- ◯ 17 weeks
- ◯ 18 weeks
- ◯ 19 weeks
- ◯ 20 weeks
- ◯ 21 weeks
- ◯ 22 weeks
- ◯ 23 weeks
- ◯ 24 weeks
- ◯ 25 weeks
- ◯ 26 weeks
- ◯ 27 weeks
- ◯ 28 weeks
- ◯ 29 weeks
- ◯ 30 weeks

Including this pregnancy, how many times have you been pregnant? Please count all pregnancies, including pregnancies ending in live births, miscarriages, stillbirths, ectopic pregnancies, and other outcomes.	◯ Once<br>◯ Twice<br>◯ Three times<br>◯ Four times<br>◯ Five times<br>◯ Six or more times
How many children do you currently have?	◯ 0<br>◯ 1<br>◯ 2<br>◯ 3<br>◯ 4<br>◯ 5<br>◯ 6 or more
Have you ever smoked cigarettes regularly, and by regularly I mean one or more cigarettes every day for at least a month?	◯ Yes<br>◯ No<br>◯ Don't know<br>◯ Prefer not to answer
At this time, are you smoking cigarettes regularly, I mean one or more cigarettes every day?	◯ Yes<br>◯ No<br>◯ Don't know<br>◯ Prefer not to answer
Have you ever had alcoholic beverages, like beer, wine, or liquor including gin, whiskey, run, or mixed drinks?	◯ Yes<br>◯ No<br>◯ Don't know<br>◯ Prefer not to answer
At this time, do you drink any alcoholic beverages, like beer, wine, or liquor including gin, whiskey, rum, or mixed drinks?	◯ Yes<br>◯ No<br>◯ Don't know<br>◯ Prefer not to answer
Have you had prenatal genetic testing?	◯ Yes<br>◯ No<br>◯ Unsure<br>◯ Prefer not to answer
Have your children gone through newborn screening?	◯ Yes<br>◯ No<br>◯ Unsure<br>◯ Prefer not to answer
Have you or anyone in your family ever had a genetic test to predict or diagnose a disease or condition?	◯ Yes<br>◯ No<br>◯ Don't know
Has anyone in your family ever had a genetic test to predict how well a medication may work for them or their risk for having a side effect?	◯ Yes<br>◯ No<br>◯ Don't know
In the last month, how frequently have you looked for information about ways to stay healthy or get better?	◯ Daily<br>◯ Weekly<br>◯ Less than weekly<br>◯ Once this month<br>◯ Never

Where do you get your health information from? Please check all that apply

☐ Doctor
☐ Other people who have similar health issues or faced the same decisions
☐ Scientific journals
☐ Internet
☐ Magazines or newspapers
☐ Other
☐ No one-I already know what I need to know

How often do you look for health information online?

◯ Daily
◯ Several times a week
◯ Weekly
◯ Monthly

How frequently do you access your My Health at Vanderbilt Patient Portal?

◯ Never
◯ Daily
◯ Several times a week
◯ Weekly
◯ Monthly

Are there things about your health that you would prefer not to know?

◯ Yes
◯ No
◯ Don't know/Choose not to answer

The following questions ask about your medications, your experience while taking them, and your opinion on medications in general.

How many different medications do you regularly take/use?

◯ None
◯ 1
◯ 2
◯ 3
◯ 4
◯ 5 or more

Medication 1 ______________________________

Medication 2 ______________________________

Medication 3 ______________________________

Medication 4 ______________________________

Medication 5 ______________________________

Medication 6 ______________________________

Medication 7 ______________________________

Medication 8 ______________________________

Medication 9

____

Medication 10

____

Please list any other medications you regularly take/use

____

A "side effect" is a secondary problem or reaction caused by taking medication. Have you ever had any type of side effect from a medication that you have been prescribed?

- ◯ Yes
- ◯ No
- ◯ Don't know

Have you ever had a side effect from medication that required immediate medical attention?

- ◯ Yes
- ◯ No
- ◯ Don't know

The last time you experienced a side effect from a medication, how much did that side effect bother you?

- ◯ Not at all bothered
- ◯ Not very bothered
- ◯ Somewhat bothered
- ◯ Very bothered
- ◯ Extremely bothered

Have you ever stopped taking a prescription medication because of the side effects?

- ◯ Yes
- ◯ No
- ◯ Don't know

Was the decision to stop taking the medication your own choice or based on doctor's recommendations?

- ◯ Your choice
- ◯ Based on doctor's recommendations
- ◯ Both your choice and based on your doctor's recommendations

Have you every stopped taking a prescription medication because it was not helping your condition?

- ◯ Yes
- ◯ No
- ◯ Don't know

Was the decision to stop taking the medication your own choice or based on doctor's recommendations?

- ◯ My own choice
- ◯ Based on doctor's recommendations
- ◯ Both my choice and based on doctor's recommendations

To your knowledge has any member of your immediate family (parents, siblings, children) ever had any side effects from a prescription medication?

- ◯ Yes
- ◯ No
- ◯ Don't know

Please state your level of agreement with each of the statements below:

	Strongly disagree	Disagree	Neither agree nor disagree	Agree	Strongly agree
My health and future will depend on my medications	◯	◯	◯	◯	◯

My health, at present, depends on my medications	◯	◯	◯	◯	◯
Without my medications, I would be very ill	◯	◯	◯	◯	◯
My medications protect me from becoming worse	◯	◯	◯	◯	◯
My life would be impossible without my medications	◯	◯	◯	◯	◯
Having to take medications worries me	◯	◯	◯	◯	◯
I sometimes worry about the long-term effects of my medications	◯	◯	◯	◯	◯
I sometimes worry about becoming too dependent on my medications	◯	◯	◯	◯	◯
My medications are a mystery to me	◯	◯	◯	◯	◯
My medications disrupt my life	◯	◯	◯	◯	◯

Please state your level of agreement with each of the statements below:

	Strongly disagree	Disagree	Neither agree nor disagree	Agree	Strongly agree
People who take medications should stop their treatment for a while every now and again	◯	◯	◯	◯	◯
Most medications are addictive	◯	◯	◯	◯	◯
Medications do more harm than good	◯	◯	◯	◯	◯
All medications are poisons	◯	◯	◯	◯	◯
Natural remedies are safer than medications	◯	◯	◯	◯	◯
Doctors place too much trust on medications	◯	◯	◯	◯	◯
Doctors use too many medications	◯	◯	◯	◯	◯
If doctors had more time with patients they would prescribe fewer medications	◯	◯	◯	◯	◯

This next set of questions ask about your experience taking your medication

	Yes	No
Do you sometimes forget to take your medication?	◯	◯
People sometimes miss taking their medications for reasons other than forgetting. Thinking over the past 2 weeks, were there any days when you did not take your medications?	◯	◯
When you travel or leave home, do you sometimes forget to bring along your medications?	◯	◯
Did you take all your medications yesterday?	◯	◯
Taking medications every day might be a real inconvenience for some people. Do you ever feel hassled about sticking to your treatment plan?	◯	◯
When you feel like your symptoms are under control, do you sometimes stop taking your medication?	◯	◯
Have you ever cut back or stopped taking your medication without telling your doctor because you felt worse when you took it?	◯	◯

Doctors often give specific directions when prescribing medication, including the number of pills to take and the time of day to take them. How often do you take your medications as directed or prescribed by your doctor?

◯ Never/rarely
◯ Once in a while
◯ Sometimes
◯ Usually
◯ All of the time

How often do you have difficulty remembering to take all of your medication?

◯ Never/rarely
◯ Once in a while
◯ Sometimes
◯ Usually
◯ All the time

The following questions are related to your decision to participate in the study.

Please indicate which, if any, of the following factors you considered in your decision to participate in this study and the level of importance of that reason in your decision:

	Major factor in my decision	Minor factor in my decision	Considered, but was not a factor in my decision	Did not consider
Your familiarity (knowledge) of the medication	○	○	○	○
Helpfulness in optimizing my medication treatment	○	○	○	○
Family history of side effects from medications or not responding to medications	○	○	○	○
Recommendations from your doctor	○	○	○	○
Your understanding about how the test will help your doctor choose the best medication for you	○	○	○	○
Concern about having a genetic test ordered and reported by a non-genetics professional	○	○	○	○
Concern about being prescribed a more expensive medication based on test results	○	○	○	○
Length of time you'll need to take the prescribed medication (e.g., 1 week vs. indefinitely)	○	○	○	○
Concern about the privacy of my test results	○	○	○	○
Worry about the possibility of side effects or needing to get another medication prescribed if this one didn't work	○	○	○	○
Providing a DNA sample for testing	○	○	○	○
Time to wait to learn of my test results	○	○	○	○
My family's opinion on genetic testing	○	○	○	○
Trust in test results	○	○	○	○
The affect the test results could have on my family	○	○	○	○
My ability to cope with the results	○	○	○	○

The following questions will help us understand your relationship with your healthcare provider. You do not have to answer any questions that makes you uncomfortable.

These next questions are about your relationship with your primary health care provider. How is your provider at...

	Poor	Fair	Good	Very good	Excellent
Being interested in you as a whole person (ask/knowing relevant details about your life and your situation; not treating you as "just a number")?	○	○	○	○	○
Explaining things clearly (fully answering your questions; explaining clearly; giving you adequate information; not being vague)?	○	○	○	○	○
Making a plan of action with you (discussing the options; involving you in decisions as much as you want to be involved; not ignoring your views)?	○	○	○	○	○

Please state your level of agreement with the following questions:

	Strongly disagree	Disagree	Neither agree nor disagree	Agree	Strongly agree
I am comfortable with my provider recommending pharmacogenetic testing	○	○	○	○	○
I am comfortable with my provider sharing results of pharmacogenetic testing	○	○	○	○	○
I am comfortable with my pharmacist having access to my pharmacogenetic test results	○	○	○	○	○
I would be comfortable with my pharmacogenetic test results being available in my medical record for all my healthcare providers to see	○	○	○	○	○
I would prefer a health care provider ask my permission before having access to my pharmacogenetic test results	○	○	○	○	○
I am confident in my provider's understanding of pharmacogenetic testing	○	○	○	○	○

The following questions will help us understand what you know about pharmacogenetic testing and your opinions on its usefulness.

How familiar are you with the following words or phrases?

	Not at all familiar	Slightly familiar	Somewhat familiar	Moderately familiar	Extremely familiar
DNA	◯	◯	◯	◯	◯
Chromosome	◯	◯	◯	◯	◯
Gene	◯	◯	◯	◯	◯
Hereditary	◯	◯	◯	◯	◯
Genomics	◯	◯	◯	◯	◯
Genetic testing	◯	◯	◯	◯	◯
Metabolizer status	◯	◯	◯	◯	◯
Precision medicine	◯	◯	◯	◯	◯
Personalized medicine	◯	◯	◯	◯	◯
Pharmacogenetics	◯	◯	◯	◯	◯

Pharmacogenetics is the use of differences in your DNA to choose the right medication or dose for you. How would you describe your understanding of how pharmacogenetic testing can be used in healthcare? Would you say you understand it...

- ◯ Very well
- ◯ Somewhat well
- ◯ A little
- ◯ Not at all

How much do you think your genes contribute to how you respond to a prescribed medication?

- ◯ A lot
- ◯ Somewhat
- ◯ A little
- ◯ Not at all
- ◯ Don't know

The following questions are about your views of pharmacogenetic testing and what you expect to gain from this study

	Strongly disagree	Disagree	Neutral	Agree	Strongly agree
Genes have a lot to do with how healthy people are	◯	◯	◯	◯	◯
I expect to learn something new about my health from this research study	◯	◯	◯	◯	◯
I can use the results from this research project to improve my health	◯	◯	◯	◯	◯
I can use the results from this research project to improve my family's health	◯	◯	◯	◯	◯
I think it is important for me to know as much as I can about my health	◯	◯	◯	◯	◯

Results from pharmacogenetic research should be put in my medical record so that my doctors can use them	○	○	○	○	○
Experts should decide which research results are returned to me	○	○	○	○	○
It is important that my health care provider tells me about these pharmacogenetic tests, before any of them are done	○	○	○	○	○
If these pharmacogenetic tests were part of my usual check-up (blood work, vision examination, etc.), it is important that my health care provider seek separate consent (i.e., approval) from me specifically for the pharamcogenetic test	○	○	○	○	○
It is not useful to take pharmacogenetic tests anyway, because my family doctor doesn't know enough to use my test results	○	○	○	○	○
It is not useful to take pharmacogenetic tests anyway, because medical specialists don't know enough to use my test results	○	○	○	○	○
If I had to pay for the pharmacogenetic tests myself, financial cost would be one of my concerns for taking these tests	○	○	○	○	○

How important are the following to you when making a decision about getting a pharmacogenetic test?

	Not important	Somewhat important	Moderately important	Very important	Extremely important
Trust in my doctor, nurse practitioner, or other healthcare provider	○	○	○	○	○
Cost of the test	○	○	○	○	○
Potential effect of the test results on my insurance eligibility	○	○	○	○	○

Potential effect of the test results on my employment	◯	◯	◯	◯	◯
Receiving payment for my blood or tissue donation (if my donation will be used for research or profit)	◯	◯	◯	◯	◯
Receiving the results of my pharmacogenetic test(s)	◯	◯	◯	◯	◯
Receiving counseling about my test results	◯	◯	◯	◯	◯
That my results will be kept private	◯	◯	◯	◯	◯

Do you plan to share the results of the pharmacogenetic test with anyone?

- ☐ Yes, with everyone
- ☐ Yes, with my healthcare provider(s)
- ☐ Yes, with family members
- ☐ Yes, with other individuals
- ☐ No

Who else do you plan to share your results with?

____

If you indicated you have not and do not plan to disclose your pharmacogenetic test results to any of your family members, please choose the reasons for not disclosing it. Check all the apply.

- ☐ Genetic information is private and personal.
- ☐ I do not have a good relationship with my family members.
- ☐ The information will not contribute to my family members health.
- ☐ The information might hurt my family members mental wellbeing.
- ☐ My family members have the right not to know.
- ☐ The information might hurt my family members change to get married.
- ☐ The information might hurt my family members reputation in the community.
- ☐ The information might hurt my family members ability to obtain insurance
- ☐ Other

Are there things about your genes you prefer not to know?

- ◯ Yes
- ◯ No
- ◯ Don't know/prefer not to answer

Please state your level of agreement with the following statements:

	Strongly disagree	Disagree	Neither disagree nor agree	Agree	Strongly agree
Learning about my pharmacogenetic test results will help my doctor and I make decisions about my treatment	◯	◯	◯	◯	◯

Pharmacogenetic testing would help guide dosing	○	○	○	○	○
Pharmacogenetic testing should be performed routinely	○	○	○	○	○
Pharmacogenetic testing would lessen the likelihood that I experience side effects from my medication	○	○	○	○	○
Learning my pharmacogenetic test results will let me know more about my children's medication	○	○	○	○	○
Learning about my test results will help me live a better life	○	○	○	○	○
Pharmacogenetic testing will help my doctor decide which medication is most likely to treat my illness	○	○	○	○	○
Pharmacogenetic testing will help my doctor decide what strength or dose of medication would work best or be safest for me	○	○	○	○	○
Pharmacogenetic testing will help me learn why I or my family member does not tolerate or respond to certain medication	○	○	○	○	○

Thank you for answering the survey questions! You are almost finished. These last questions ask how you will get your blood drawn and confirm the email address where your Amazon gift card will be sent after you give the blood sample.

Are you scheduled to see a VUMC provider or have your blood drawn at a VUMC lab or clinic visit in the next 3 months?

○ Yes
○ No

To respect your time and save you a trip, we will put a request in the computer system to have your blood drawn for this study at a VUMC lab. It will take 3 - 5 business days to put the request into the system. The request will be valid for 3 months.

If you are already planning to have your blood drawn at a VUMC lab for an upcoming clinic visit in the next 3 months, an extra tube will be taken for the study at that time.

If a blood draw is not part of your clinic or lab visit, you may go a VUMC lab of your choice to get your blood drawn in the next 3 months.

We will send a follow-up email after you complete this survey to give you a list of VUMC labs with their locations. Please wait until you receive the email before you go to a lab.

To respect your time, we will put a request in the computer system to have your blood drawn for this study at a VUMC lab. It will take 3 -5 business days to put the request into the system. The request will be valid for 3 months.

You can go to the lab in the next 3 months on a day that works for you. You do not need an appointment.

We will send a follow-up email after you complete this survey to give you a list of VUMC labs with their locations. Please wait until you receive the email before you go to a lab.

After you provide a blood sample, we will send you a $20 Amazon gift card by email. You will receive other $5 gift cards for completing later surveys, up to a possible maximum of $30 for some participants.

Please confirm the email address you would like us to send the gift card to: ________________________________

If a gift card will not work for you, please contact the study by email at: pgx-survey@vumc.org or by phone at 615-875-4491.

Thank you for participating in the MPRINT Study!
