# Supplementary material for: A randomized trial to evaluate attitudes regarding pharmacogenomics among pregnant and pediatric populations: design and baseline characteristics
Source: Pharmacogenomics J. 2026 Apr 23;26(3):16. doi: 10.1038/s41397-026-00413-5 (PMC13106030; doi:10.1038/s41397-026-00413-5)
Supplement: Supplementary file 4 — Appendix 4 [file 41397_2026_413_MOESM4_ESM.pdf]

Pediatric Baseline Survey

Please complete the survey below.

Thank you!

For this survey, the study PARTICIPANT is the infant, child, or adolescent enrolled in the study. We will refer to them as "your child" throughout the survey. We ask that the parent or guardian complete these surveys, with input from their child whenever possible.

Who is completing this survey?

- ◯ The parent/guardian only
- ◯ The parent/guardian with input from the participant

The next few questions will ask for your contact information and how you would like us to stay in touch.

First Name ______________________________

Last Name ______________________________

Street Address ______________________________

City ______________________________

State ______________________________

Zip Code ______________________________

The following questions will help us describe who participates in the study. You do not have to answer questions that make you feel uncomfortable.

What is your child's ethnicity?

- ◯ Hispanic or Latino
- ◯ NOT Hispanic or Latino
- ◯ Unknown
- ◯ Prefer not to answer

What is your child's race? Please check all that apply.

- ☐ American Indian or Alaska Native
- ☐ Asian
- ☐ Black or African American
- ☐ Native Hawaiian or Other Pacific Islander
- ☐ White or European American
- ☐ More Than One Race
- ☐ Unknown
- ☐ Prefer not to answer

What sex was your child assigned at birth, on the original birth certificate?

- ◯ Female
- ◯ Male
- ◯ Intersex
- ◯ Prefer not to answer

What is your child's current gender identity?	◯ Female<br>◯ Male<br>◯ Transgender<br>◯ Do not identify as female, male, or transgender<br>◯ Prefer not to answer
What is the highest grade your child has completed?	◯ None<br>◯ Kindergarten<br>◯ First grade<br>◯ Second grade<br>◯ Third grade<br>◯ Forth grade<br>◯ Fifth grade<br>◯ Sixth grade<br>◯ Seventh grade<br>◯ Eighth grade<br>◯ Ninth grade<br>◯ Tenth grade<br>◯ Eleventh grade<br>◯ Twelfth grade (high school graduate)<br>◯ Some post-high school training (college or occupational, technical, or vocational training)<br>◯ Prefer not to answer
What is your (parent or guardian's) current marital status?	◯ Single<br>◯ Married or living as married<br>◯ Widowed<br>◯ Divorced<br>◯ Separated<br>◯ Other<br>◯ Prefer not to answer
Including yourself, how many people currently live in your household	◯ 1<br>◯ 2<br>◯ 3<br>◯ 4<br>◯ 5<br>◯ 6<br>◯ 7<br>◯ 8<br>◯ 9<br>◯ 10+
How religious do you consider yourself to be?	◯ Not religious at all<br>◯ Not very religious<br>◯ Somewhat religious<br>◯ Very religious<br>◯ Prefer not to answer
What is your (parent or guardian's) current work situation?	◯ Working<br>◯ Retired<br>◯ Disabled (permanently or temporarily)<br>◯ Other<br>◯ Prefer not to answer

What is your household's total combined income (pre-taxes) during the past 12 months?

- ◯ Less than $25,000
- ◯ $25,000 to $50,000
- ◯ $50,000 to $75,000
- ◯ $75,000 to $100,000
- ◯ $100,000 or more
- ◯ Don't know
- ◯ Prefer not to answer

Does your child have health insurance or a health coverage plan? Please check all that apply

- ☐ Yes, through my employer
- ☐ Yes, through someone else's employer
- ☐ Yes, a plan that I or someone else buys
- ☐ Yes, through Medicare
- ☐ Yes, through Medicaid or Medical Assistance
- ☐ Yes, through the military, CHAMPUS, or the VA
- ☐ No, I don't have any coverage
- ☐ Don't know
- ☐ Prefer not to answer

How did you hear about the study?

- ☐ Vanderbilt email
- ☐ Research Match email
- ☐ Doctor or clinic told me about the study
- ☐ Letter or flyer in clinic
- ☐ Word of mouth
- ☐ Other

Other: ______________________________

The following questions will help us understand your child's health history and their family health history. If family history information is unavailable for any reason, please mark "unsure" or "don't know." You do not have to answer questions that make you uncomfortable.

How tall is your child (in feet and/or inches)? ______________________________

How much does your child currently weigh (in pounds)? ______________________________

In general, would you say your child's health is...

- ◯ Excellent
- ◯ Very good
- ◯ Good
- ◯ Fair
- ◯ Poor

Do any diseases, like heart disease, cancer, or diabetes, run in your family?

- ◯ Yes
- ◯ No
- ◯ Unsure
- ◯ Prefer not to answer

Do you think any of the diseases are caused by genes?

- ◯ Yes
- ◯ No
- ◯ Unsure
- ◯ Prefer not to answer

What disease do you believe is caused by a gene? ______________________________

Does your child have any of the following chronic conditions? Please check all that apply.

- ☐ Heart or vascular condition (Abnormal heart rhythm, congenital heart disease or other conditions affect the heart or blood vessels)
- ☐ Lung condition (Asthma, cystic fibrosis or other conditions that affect breathing)
- ☐ Gastrointestinal or liver condition (Acid reflux, Crohn's disease, Celiac disease, hepatitis, or other conditions affecting your digestion or how your liver works)
- ☐ Kidney condition (Nephrotic syndrome, congenital kidney disease, and other conditions affecting how your body makes urine)
- ☐ Neurologic condition (Seizures, headaches, sleep disorders, and other conditions that affect the brain, spinal cord, and neurons)
- ☐ Conditions affecting your immune system (Immune deficiency, HIV/AIDS and other long-term viral, bacterial, fungal or other infections)
- ☐ Rheumatologic or autoimmune condition (Juvenile arthritis, lupus, and other conditions due to the immune system)
- ☐ Endocrine condition (Thyroid disease, diabetes, and other conditions affecting hormones)
- ☐ Hematologic (blood cell) condition (Conditions affecting red blood cells (anemia, thalassemia), white blood cells, or platelets)
- ☐ Skin condition (Eczema, Atopic dermatitis, psoriasis, and other conditions affecting the skin)
- ☐ Cancer (Cancer of any type, now or in the past)
- ☐ Behavioral or mental health condition (Autism, attention deficit, speech/language disorders, depression, anxiety, and other conditions affecting mental health and behavior)
- ☐ Reproductive or gynecological condition (Any conditions affecting the male or female reproductive organs)
- ☐ Other

Please list other chronic diseases your child has

____

Have you or anyone in your family ever had a genetic test to predict or diagnose a disease or condition?

- ◯ Yes
- ◯ No
- ◯ Don't know

Has anyone in your family ever had a genetic test to predict how well a medication may work for them or their risk for having a side effect?

- ◯ Yes
- ◯ No
- ◯ Don't know

In the last month, how frequently have you looked for information about ways to stay healthy or get better?

- ◯ Daily
- ◯ Weekly
- ◯ Less than weekly
- ◯ Once this month
- ◯ Never

Where do you get your health information from? Please check all that apply	☐ Doctor<br>☐ Other people who have similar health issues or faced the same decisions<br>☐ Scientific journals<br>☐ Internet<br>☐ Magazines or newspapers<br>☐ Other<br>☐ No one-I already know what I need to know
How often do you look for health information online?	◯ Daily<br>◯ Several times a week<br>◯ Weekly<br>◯ Monthly
How frequently do you access your child's My Health at Vanderbilt Patient Portal?	◯ Never<br>◯ Daily<br>◯ Several times a week<br>◯ Weekly<br>◯ Monthly
Are there things about your child's health that you would prefer not to know?	◯ Yes<br>◯ No<br>◯ Don't know/Choose not to answer

The following questions ask about your child's medications, their experience while taking them, and your opinion on medications in general.

How many different medications does your child regularly take/use?	◯ None<br>◯ 1<br>◯ 2<br>◯ 3<br>◯ 4<br>◯ 5 or more
Medication 1	______________________________
Medication 2	______________________________
Medication 3	______________________________
Medication 4	______________________________
Medication 5	______________________________
Medication 6	______________________________
Medication 7	______________________________
Medication 8	______________________________

Medication 9

____

Medication 10

____

Please list any additional medications your child regularly takes/uses

____

A "side effect" is a secondary problem or reaction caused by taking medication. Has your child ever had any type of side effect from a medication that they have been prescribed?

- ◯ Yes
- ◯ No
- ◯ Don't know

Has your child ever had a side effect from medication that required immediate medical attention?

- ◯ Yes
- ◯ No
- ◯ Don't know

The last time your child experienced a side effect from a medication, how much did that side effect bother them?

- ◯ Not at all bothered
- ◯ Not very bothered
- ◯ Somewhat bothered
- ◯ Very bothered
- ◯ Extremely bothered

Has your child ever stopped taking a prescription medication because of the side effects?

- ◯ Yes
- ◯ No
- ◯ Don't know

Was the decision to stop taking the medication your own choice (parent/guardian and/or your child's) or based on doctor's recommendations?

- ◯ Your choice
- ◯ Based on doctor's recommendations
- ◯ Both your choice and based on your doctor's recommendations

Has your child every stopped taking a prescription medication because it was not helping their condition?

- ◯ Yes
- ◯ No
- ◯ Don't know

Was the decision to stop taking the medication your own choice (parent/guardian and/or your child's) or based on doctor's recommendations?

- ◯ My own choice
- ◯ Based on doctor's recommendations
- ◯ Both my choice and based on doctor's recommendations

To your knowledge has any member of your immediate family (parents, siblings, children) ever had any side effects from a prescription medication?

- ◯ Yes
- ◯ No
- ◯ Don't know

Please state your level of agreement with each of the statements below:

	Strongly disagree	Disagree	Neither agree nor disagree	Agree	Strongly agree
My child's health and future will depend on their medications	◯	◯	◯	◯	◯

My child's health, at present, depends on their medications	◯	◯	◯	◯	◯
Without my child's medications, they would be very ill	◯	◯	◯	◯	◯
My child's medications protect them from becoming worse	◯	◯	◯	◯	◯
My child's life would be impossible without their medications	◯	◯	◯	◯	◯
My child having to take medications worries me	◯	◯	◯	◯	◯
I sometimes worry about the long-term effects of my child's medications	◯	◯	◯	◯	◯
I sometimes worry about my child becoming too dependent on their medications	◯	◯	◯	◯	◯
My child's medications are a mystery to me	◯	◯	◯	◯	◯
My child's medications disrupt my life	◯	◯	◯	◯	◯

Please state your level of agreement with each of the statements below:

	Strongly disagree	Disagree	Neither agree nor disagree	Agree	Strongly agree
People who take medications should stop their treatment for a while every now and again	◯	◯	◯	◯	◯
Most medications are addictive	◯	◯	◯	◯	◯
Medications do more harm than good	◯	◯	◯	◯	◯
All medications are poisons	◯	◯	◯	◯	◯
Natural remedies are safer than medications	◯	◯	◯	◯	◯
Doctors place too much trust on medications	◯	◯	◯	◯	◯
Doctors use too many medications	◯	◯	◯	◯	◯
If doctors had more time with patients they would prescribe fewer medications	◯	◯	◯	◯	◯

This next set of questions ask about your child's experience taking their medication

	Yes	No
Does your child sometimes forget to take their medication?	◯	◯
People sometimes miss taking their medications for reasons other than forgetting. Thinking over the past 2 weeks, were there any days when your child did not take their medications?	◯	◯
When you travel or leave home with your child, does your child sometimes forget to bring along their medications?	◯	◯
Did your child take all their medications yesterday?	◯	◯
Taking medications every day might be a real inconvenience for some people. Do you ever feel hassled about sticking to your child's treatment plan?	◯	◯
When your child feels like their symptoms are under control, does your child sometimes stop taking their medication?	◯	◯
Has your child ever cut back or stopped taking their medication without telling their doctor because they felt worse when they took it?	◯	◯

Doctors often give specific directions when prescribing medication, including the number of pills to take and the time of day to take them. How often does your child take their medications as directed or prescribed by their doctor?

◯ Never/rarely
◯ Once in a while
◯ Sometimes
◯ Usually
◯ All of the time

How often does your child have difficulty remembering to take all of their medication?

◯ Never/rarely
◯ Once in a while
◯ Sometimes
◯ Usually
◯ All the time

The following questions are related to your decision to participate in the study.

Please indicate which, if any, of the following factors you considered in your decision to participate in this study and the level of importance of that reason in your decision:

	Major factor in my decision	Minor factor in my decision	Considered, but was not a factor in my decision	Did not consider
Your familiarity (knowledge) of the medication	◯	◯	◯	◯
Helpfulness in optimizing your child's medication treatment	◯	◯	◯	◯
Family history of side effects from medications or not responding to medications	◯	◯	◯	◯
Recommendations from your child's doctor	◯	◯	◯	◯
Your understanding about how the test will help your child's doctor choose the best medication for them	◯	◯	◯	◯
Concern about having a genetic test ordered and reported by a non-genetics professional	◯	◯	◯	◯
Concern about being prescribed a more expensive medication based on test results	◯	◯	◯	◯
Length of time your child need to take the prescribed medication (e.g., 1 week vs. indefinitely)	◯	◯	◯	◯
Concern about the privacy of your child's test results	◯	◯	◯	◯
Worry about the possibility of side effects or needing to get another medication prescribed if this one didn't work	◯	◯	◯	◯
Providing a DNA sample for testing	◯	◯	◯	◯
Time to wait to learn of my child's test results	◯	◯	◯	◯
My family's opinion on genetic testing	◯	◯	◯	◯
Trust in test results	◯	◯	◯	◯
The affect the test results could have on my family	◯	◯	◯	◯
My ability to cope with the results	◯	◯	◯	◯

The following questions will help us understand your relationship with your child's healthcare provider. You do not have to answer any questiosn that makes you uncomfortable.

These next questions are about your relationship with your child's primary health care provider. How is your child's provider at...

	Poor	Fair	Good	Very good	Excellent
Being interested in your child as a whole person (ask/knowing relevant details about their life and their situation; not treating them as "just a number")?	○	○	○	○	○
Explaining things clearly (fully answering your questions; explaining clearly; giving you adequate information; not being vague)?	○	○	○	○	○
Making a plan of action with you (discussing the options; involving you in decisions as much as you want to be involved; not ignoring your views)?	○	○	○	○	○

Please state your level of agreement with the following questions:

	Strongly disagree	Disagree	Neither agree nor disagree	Agree	Strongly agree
I am comfortable with my child's provider recommending pharmacogenetic testing	○	○	○	○	○
I am comfortable with my child's provider sharing results of pharmacogenetic testing	○	○	○	○	○
I am comfortable with my child's pharmacist having access to their pharmacogenetic test results	○	○	○	○	○
I would be comfortable with my child's pharmacogenetic test results being available in their medical record for all their healthcare providers to see	○	○	○	○	○
I would prefer a health care provider ask my permission before having access to my child's pharmacogenetic test results	○	○	○	○	○

I am confident in my child's provider's understanding of pharmacogenetic testing	◯	◯	◯	◯	◯

The following questions will help us understand what you know about pharmacogenetic testing and your opinions on its usefulness.

How familiar are you with the following words or phrases?

	Not at all familiar	Slightly familiar	Somewhat familiar	Moderately familiar	Extremely familiar
DNA	◯	◯	◯	◯	◯
Chromosome	◯	◯	◯	◯	◯
Gene	◯	◯	◯	◯	◯
Hereditary	◯	◯	◯	◯	◯
Genomics	◯	◯	◯	◯	◯
Genetic testing	◯	◯	◯	◯	◯
Metabolizer status	◯	◯	◯	◯	◯
Precision medicine	◯	◯	◯	◯	◯
Personalized medicine	◯	◯	◯	◯	◯
Pharmacogenetics	◯	◯	◯	◯	◯

Pharmacogenetics is the use of differences in your DNA to choose the right medication or dose for you. How would you describe your understanding of how pharmacogenetic testing can be used in healthcare? Would you say you understand it...

- ◯ Very well
- ◯ Somewhat well
- ◯ A little
- ◯ Not at all

How much do you think your child's genes contribute to how they respond to a prescribed medication?

- ◯ A lot
- ◯ Somewhat
- ◯ A little
- ◯ Not at all
- ◯ Don't know

The following questions are about your views of pharmacogenetic testing and what you expect to gain from this study

	Strongly disagree	Disagree	Neutral	Agree	Strongly agree
Genes have a lot to do with how healthy people are	◯	◯	◯	◯	◯
I expect to learn something new about my child's health from this research study	◯	◯	◯	◯	◯
I can use the results from this research project to improve my child's health	◯	◯	◯	◯	◯

I can use the results from this research project to improve my family's health	○	○	○	○	○
I think it is important for me to know as much as I can about my child's health	○	○	○	○	○
Results from pharmacogenetic research should be put in my child's medical record so that their doctors can use them	○	○	○	○	○
Experts should decide which research results are returned to me	○	○	○	○	○
It is important that my child's health care provider tells me about these pharmacogenetic tests, before any of them are done	○	○	○	○	○
If these pharmacogenetic tests were part of my child's usual check-up (blood work, vision examination, etc.), it is important that my child's health care provider seek separate consent (i.e., approval) from me specifically for the pharamcogenetic test	○	○	○	○	○
It is not useful to take pharmacogenetic tests anyway, because my child's doctor doesn't know enough to use their test results	○	○	○	○	○
It is not useful to take pharmacogenetic tests anyway, because medical specialists don't know enough to use my child's test results	○	○	○	○	○
If I had to pay for pharmacogenetic tests myself, financial cost would be one of my concerns for taking these tests	○	○	○	○	○

How important are the following to you when making a decision about getting a pharmacogenetic test?

	Not important	Somewhat important	Moderately important	Very important	Extremely important
Trust in my doctor, nurse practitioner, or other healthcare provider	○	○	○	○	○
Cost of the test	○	○	○	○	○
Potential effects of the test results on your child's insurance eligibility	○	○	○	○	○
Receiving payment for my blood or tissue donation (if my donation will be used for research or profit)	○	○	○	○	○
Receiving the results of my pharmacogenetic test(s)	○	○	○	○	○
Receiving counseling about my test results	○	○	○	○	○
That my results will be kept private	○	○	○	○	○

Do you plan to share the results of your child's pharmacogenetic test with anyone?

- ☐ Yes, with my child
- ☐ Yes, with everyone
- ☐ Yes, with my healthcare provider(s)
- ☐ Yes, with family members
- ☐ Yes, with other individuals
- ☐ No

Who else do you plan to share your child's results with?

____

If you indicated you have not and do not plan to disclose your child's pharmacogenetic test results to any of your family members, please choose the reasons for not disclosing it. Check all the apply.

- ☐ Genetic information is private and personal.
- ☐ I do not have a good relationship with my family members.
- ☐ The information will not contribute to my family members health.
- ☐ The information might hurt my family members mental wellbeing.
- ☐ My family members have the right not to know.
- ☐ The information might hurt my family members change to get married.
- ☐ The information might hurt my family members reputation in the community.
- ☐ The information might hurt my family members ability to obtain insurance
- ☐ Other

Are there things about your child's genes you prefer not to know?

- ○ Yes
- ○ No
- ○ Don't know/prefer not to answer

Please state your level of agreement with the following statements:

	Strongly disagree	Disagree	Neither disagree nor agree	Agree	Strongly agree
Learning about my child's pharmacogenetic test results will help their doctor and I make decisions about their treatment	○	○	○	○	○
Pharmacogenetic testing would help guide dosing	○	○	○	○	○
Pharmacogenetic testing should be performed routinely	○	○	○	○	○
Pharmacogenetic testing would lessen the likelihood that my child experiences side effects from their medication	○	○	○	○	○
Learning my child's pharmacogenetic test results will let me know more about my their medication	○	○	○	○	○
Learning about my child's test results will help them live a better life	○	○	○	○	○
Pharmacogenetic testing will help my child's doctor decide which medication is most likely to treat their illness	○	○	○	○	○
Pharmacogenetic testing will help my child's doctor decide what strength or dose of medication would work best or be safest for them	○	○	○	○	○
Pharmacogenetic testing will help me learn why my child or my family member does not tolerate or respond to certain medication	○	○	○	○	○

Thank you for answering the survey questions! You are almost finished. These last questions ask confirm the email address where the Amazon gift card will be sent after your child gives the blood sample.

After your child provides a blood sample, we will send a $20 Amazon gift card by email. You will receive other $5 gift cards for completing later surveys, up to a possible maximum of $30 for some participants.

Please confirm the email address you would like us to send the gift card to: ______________________________

If a gift card will not work for you, please contact the study by email at: pgx-survey@vumc.org or by phone at 615-875-4491.

Thank you for participating in the MPRINT Study!
