# Supplementary material for: A randomized trial to evaluate attitudes regarding pharmacogenomics among pregnant and pediatric populations: design and baseline characteristics
Source: Pharmacogenomics J. 2026 Apr 23;26(3):16. doi: 10.1038/s41397-026-00413-5 (PMC13106030; doi:10.1038/s41397-026-00413-5)
Supplement: Supplementary file 5 — Appendix 5 [file 41397_2026_413_MOESM5_ESM.docx]

**Appendix 5:** Pharmacogenetic Testing Panel

| **Genetic Variants** | **Medications with PGx Based Clinical Decision Support** |
| --- | --- |
| *DPYD* c.1905+1G>A (*2, rs3918290), c.1679T>G (*13, rs55886062), c.557A>G, c.1236G>A (HapB3, rs56038477), c.2846A>T (rs67376798)(NM_000110) | Capecitabine, fluorouracil |
| *CYP2C19* *2 (rs4244285), *3 (rs4986893), *4 (rs28399504), *6 (rs72552267), *8 (rs41291556), *17 (rs12248560) | Clopidogrel, escitalopram, citalopram, sertraline, voriconazole |
| *CYP2C9* *2 (rs1799853), *3 (rs1057910), *6 (rs9332131), *8 (rs7900194), *11 (rs28371685) | Warfarin |
| *CYP2D6**2 (rs16947), *3 (rs35742686), *4 (rs3892097), *6 (rs5030655), *7 (rs5030867), *9 (rs5030656), *10 (rs1065852), *15 (rs774671100), *17 (rs28371706), *29 (rs59421388), *41 (rs28371725), *59 (rs79292917) | Codeine, fluvoxamine, ondansetron, paroxetine, tramadol |
| *CYP3A5**3 (rs776746), *6 (rs10264272), *7 (rs41303343) | Tacrolimus |
| *CYP4F2* *3 (rs2108622) | Warfarin |
| *NUDT15**2 or *3 (rs116855232), *5 (rs186364861) | Thiopurines (mercaptopurine, azathioprine and thioguanine) |
| *SLCO1B1**5 (rs4149056) | Simvastatin |
| *TPMT* *2 (rs1800462), *3A (inferred based on rs1800460 and rs1142345), 3B (rs1800460), *3C (rs1142345), *4 (rs1800584), *8 (rs56161402) | Thiopurines (mercaptopurine, azathioprine and thioguanine) |
| *VKORC1* c.-1639G>A (NM_024006) | Warfarin |

**References**

1. Vnencak-Jones CL, Saucier LAG, Liu M, Gatto CL, Peterson JF. Pharmacogenomics: Genotype-Driven Medicine. J Appl Lab Med. 2024 Jan 3;9(1):183-186. doi: 10.1093/jalm/jfad064. PMID: 38167767.
