# Supplementary material for: A randomized trial to evaluate attitudes regarding pharmacogenomics among pregnant and pediatric populations: design and baseline characteristics
Source: Pharmacogenomics J. 2026 Apr 23;26(3):16. doi: 10.1038/s41397-026-00413-5 (PMC13106030; doi:10.1038/s41397-026-00413-5)
Supplement: Supplementary file 6 — Appendix 6 [file 41397_2026_413_MOESM6_ESM.docx]

**Appendix 6:** VICE-MPRINT: Maternal and Pediatric Pharmacogenetics Survey

Welcome to our Education Video!

This video is about:

- a specific type of genetic testing, called pharmacogenetic testing
- how it works, and
- how it can help doctors prescribe the correct medication, at the right doses, specifically for you.

Genes are the DNA instructions you inherit from your mother and your father.

Different types of genetic testing, or DNA testing, look at different genes. The kind that looks at your genes to **predict how medications will work** is called **pharmacogenetic testing**. This kind of test might:

- assess how your body will process (or metabolize) the medication,
- determine if you might have side effects from taking a medication, and
- help choose the best treatment for you

Pharmacogenetic test results are just one piece of information that will help your doctor make a good decision about what dose of a medication is best for you. There are also other things that affect how medications work in the body, like changes that happen during childhood and puberty and changes that happen during pregnancy.

Some people process or metabolize a medication really fast while others metabolize it more slowly. That is one reason why one medication can work great for one person, but cause side effects, or not work at all, for another. Pharmacogenetic testing can help predict how well a medication may work for you, and how much of the medication would be best.

Not ALL medications currently have a pharmacogenetic test, but we DO have testing available for several medications that are used to treat some common problems such as:

- depression
- heartburn
- blood clots
- pain
- high cholesterol
- nausea, and
- cancer

A few days after you provide your sample for pharmacogenetic testing, your results will be available at www.myhealthatvanderbilt.com:

- Click on “Your Menu”
- Then scroll down and click on “Genetic Profile”

Your Genetic Profile will have two sections:

- The “Genetic findings of interest” on top of the page that may require more attention
- And “Other Genetic Findings” on the bottom section of the page.

You can also find information about medications that may be relevant to you, either now or if you ever need these medications in the future. There will be a short description of the medication, and your test result. Click on any of the text boxes or on “More information” to learn about the medication and the gene interaction.

Finally, pharmacogenetic tests let doctors make a prediction of how a person will process (or metabolize) a medication. This prediction is called a Metabolizer Status. The metabolizer status can range from:

- “Poor” (meaning certain medications are processed very slowly or not at all), to
- “Ultrarapid” (meaning certain medications are processed very fast)

If a medication is processed or metabolized too slowly or too fast, it can increase the chances of having side effects, or of it not working at all. In this example, the patient is an ultrarapid metabolizer of citalopram, also known as Celexa.

Because of this, their doctor might choose a different medication that is metabolized differently. For some circumstances, the doctor may just change the dose of the medication.

Doctors at Vanderbilt will see the results in the electronic health record. And if you have questions about the test results, you can message your doctor through the My Health at Vanderbilt website.

If you would like to share the results with doctors outside of Vanderbilt, bring a copy of the lab report to your next appointment.

Remember, pharmacogenetic results are one piece of the puzzle. Don't change any medications or doses without speaking to your doctor first.

Thanks for watching!
