# Supplementary material for: A randomized trial to evaluate attitudes regarding pharmacogenomics among pregnant and pediatric populations: design and baseline characteristics
Source: Pharmacogenomics J. 2026 Apr 23;26(3):16. doi: 10.1038/s41397-026-00413-5 (PMC13106030; doi:10.1038/s41397-026-00413-5)
Supplement: Supplementary file 7 — Appendix 7 [file 41397_2026_413_MOESM7_ESM.docx]

**Appendix 7.** Power calculation

Assuming equal randomization and an α=0.05, we will have 80% power to detect and effectiveness of study intervention in participant-reported improved understanding of PGx testing for a risk ratio of a magnitude of 1.35 or higher.
